# Boosting neuregulin 1 type-III expression hastens SMA motor axon maturation

**DOI:** 10.1186/s40478-023-01551-8

**Published:** 2023-03-30

**Authors:** Lingling Kong, Cera W. Hassinan, Florian Gerstner, Jannik M. Buettner, Jeffrey B. Petigrow, David O. Valdivia, Michelle H. Chan-Cortés, Amy Mistri, Annie Cao, Scott Alan McGaugh, Madeline Denton, Stephen Brown, Joshua Ross, Markus H. Schwab, Christian M. Simon, Charlotte J. Sumner

**Affiliations:** 1 Departments of Neurology, Johns Hopkins University School of Medicine, 855 North Wolfe Street, Rangos Building Room 234, Baltimore, MD 21205 USA; 2 Carl-Ludwig-Institute for Physiology, Leipzig University, Leipzig, Germany; 3 Department of Neuropathology, University Hospital Leipzig, Leipzig, Germany; 4 Departments of Neuroscience, Johns Hopkins University School of Medicine, Baltimore, MD 21205 USA

**Keywords:** Spinal muscular atrophy (SMA), Motor axon, Neuregulin 1 type III (NRG1-III), Conduction velocity, Neuromuscular junction (NMJ)

## Abstract

Intercellular communication between axons and Schwann cells is critical for attaining the complex morphological steps necessary for axon maturation. In the early onset motor neuron disease spinal muscular atrophy (SMA), many motor axons are not ensheathed by Schwann cells nor grow sufficiently in radial diameter to become myelinated. These developmentally arrested motor axons are dysfunctional and vulnerable to rapid degeneration, limiting efficacy of current SMA therapeutics. We hypothesized that accelerating SMA motor axon maturation would improve their function and reduce disease features. A principle regulator of peripheral axon development is neuregulin 1 type III (NRG1-III). Expressed on axon surfaces, it interacts with Schwann cell receptors to mediate axon ensheathment and myelination. We examined NRG1 mRNA and protein expression levels in human and mouse SMA tissues and observed reduced expression in SMA spinal cord and in ventral, but not dorsal root axons. To determine the impact of neuronal NRG1-III overexpression on SMA motor axon development, we bred NRG1-III overexpressing mice to SMA∆7 mice. Neonatally, elevated NRG1-III expression increased SMA ventral root size as well as axon segregation, diameter, and myelination resulting in improved motor axon conduction velocities. NRG1-III was not able to prevent distal axonal degeneration nor improve axon electrophysiology, motor behavior, or survival of older mice. Together these findings demonstrate that early SMA motor axon developmental impairments can be ameliorated by a molecular strategy independent of SMN replacement providing hope for future SMA combinatorial therapeutic approaches.

**Supplementary Information:**

The online version contains supplementary material available at 10.1186/s40478-023-01551-8.

## Introduction

The motor neuron (MN) disease spinal muscular atrophy (SMA) is caused by recessive mutations of the survival motor neuron 1 (*SMN1)* gene, retention of variable copies of an alternatively spliced, paralogous gene *SMN2*, and deficient expression of the SMN protein [25, 26, 28]. Severely affected patients who have only two copies of *SMN2* experience severe muscle weakness within weeks or months of birth. Three disease modifying treatments—the antisense oligonucleotide nusinersen, the gene replacement therapy onasemnogene abeparvovec, and small molecule risdiplam—increase SMN levels, but therapeutic efficacy is limited in part by early neurodegeneration [31]. This is evidenced by measurement of neurofilament (NF) levels released into serum or cerebrospinal fluid (CSF) from axons during degeneration. Serum and CSF NFs are most elevated in severe SMA patients neonatally, even prior to detectable muscle weakness, and thereafter decline [8, 9].

In an effort to understand the cellular basis of this early degeneration, we recently characterized MNs and their axons in severe SMA patient human tissues and in a severe SMA mouse model (SMAΔ7 mice) [22]. We observed that many SMA motor axons and their surrounding Schwann cells are slowed in their development beginning in utero. In order to acquire a large myelinated axon phenotype capable of rapid conduction velocities, individual motor axons must progress through a series of morphological stages becoming individually ensheathed by Schwann cell cytoplasm, segregated 1:1 with Schwann cells after Schwann cell proliferation, and myelinated proportionate to radial diameter [13, 49]. Instead, many SMA motor axons are stalled in clusters of directly abutting, small axons less than 1 µm in diameter associated with a reduced number of Schwann cells. Although some SMA motor axons mature sufficiently to become myelinated, they are reduced in diameter and have slowed conduction velocities. These developmental impairments precede neurodegeneration with the most developmentally lagging axons vulnerable to rapid degeneration neonatally [22]. Suboptimal efficacy of current therapeutics in some SMA patients likely results from failure to reverse developmental abnormalities as well as prevent neurodegeneration. Indeed, in severe SMA mice, prenatal rather than postnatal initiation of a risdiplam analogue was required to improve the maturation and maintenance of the most developmentally impaired SMA motor axons [22]. Strategies to accelerate SMA motor axon maturation *postnatally* have the potential to prevent their early degeneration and improve their function. Recent electrophysiological studies suggest that partial improvements in axonal maturation in SMA patients treated with nusinersen are associated with gains in motor function [20, 21].

The molecular mechanisms underlying impaired motor axon maturation in SMA are unknown. The completion of a complex series of morphological steps for axon radial maturation requires bidirectional signaling between axons and Schwann cells [12, 46]. Neuregulin 1 (NRG1) is a member of an epidermal growth factor (EGF)-like family of growth factors that plays essential roles in nervous system development and nerve repair [3]. The human *NRG1* gene encodes 6 principal NRG1 isoforms via alternative splicing and differential promoter usage [7]. NRG1-III is the major isoform produced by MNs in the developing spinal cord [23, 32]. It is a membrane tethered growth factor where it interacts with ErbB2/3 receptors on Schwann cells. It is essential for Schwann cell proliferation, motility, survival, and peripheral axon ensheathment and myelination with the amount of NRG1-III determining the ensheathment fate and myelin thickness of an axon [33, 39, 47]. NRG1 also helps maintain the functional integrity and survival of peripheral nerve axons and therefore NRG1/ErbB signaling has also been considered a potential therapeutic target in neurodevelopmental and neurodegenerative diseases [40, 44].

Here, after observing that NRG1 expression was reduced in SMA human and mouse tissues, we assessed whether boosting NRG1-III expression in SMA mice could improve motor axon maturation. Importantly, NRG1-III overexpression increased SMA motor axon ensheathment and myelination in neonatal SMA mice resulting in increased conduction speed. Although NRG1-III did not prevent distal axonal degeneration nor provide long term benefit to SMA mice, this study is an important proof of principle that SMA axonal developmental phenotypes can be ameliorated with therapeutic strategies independent of SMN induction opening the possibility to future combination therapeutic strategies.

## Material and methods

### Human samples

De-identified human cervical spinal cord (CSC), ventral root (VR), and dorsal root (DR) tissues (Table 1) were collected at expedited autopsies following parental- or patient-informed consent in strict observance of legal and institutional ethical regulations as previously described [22, 42].

**Table 1:** Human tissues

Case ID	Age (months)	PMI (hours)	*SMN1 copy #*	*SMN2 copy #*	Tissue	Cause of death
CNTL 12-02	0.03	26	2	2	L3 VR, L5 DR,	Unknown
CNTL 90-08	1.3	8	2	2	CSC	Arthrogryposis
CNTL 02-02	3	15	2	2	CSC	Nemaline myopathy
CNTL 08-01	4	14.5	2	2	L4 VR, CSC	Unknown
CNTL 95-03	6	8	N/A	N/A	CSC	Megacystis microcolon
CNTL 17-01	9	14	N/A	N/A	L1 VR, L1 DR	Trisomy 21, viral pneumonia
CNTL 12-05	19	19	2	1	L3 VR, L3 DR	Unknown
SMA 11-01	1.8	7	0	2	L2 VR, L2 DR, CSC	Type 1 SMA
SMA 12-01	2.5	7	0	2	L3 VR, L2 DR, CSC	Type 1 SMA
SMA 09-02	4	4	0	2	CSC	Type 1 SMA
SMA 92-01	5.5	14	0	2	CSC	Type 1 SMA
SMA 10-14	7	25	0	2	L1 VR, L3 DR	Type 1 SMA
SMA 14-05	8	6	0	2	CSC	Type I SMA
SMA 94-06	12.8	10	0	2	CSC	Type 1 SMA
SMA 14-04	72	24	0	2	L4 VR	Type 1 SMA

PMI = post-mortem interval, CNTL = non-SMA control subject, SMA = SMA subject, CSC = cervical spinal cord, DR = dorsal root, L = lumbar, VR = ventral root, N/A = not available

### Mouse lines and assessments

Experiments were performed in accordance with the National Institutes of Health Guide for Care and Use of Laboratory Animals and approved by Institutional Animal Care and Use Committees (IACUCs) at Johns Hopkins University School of Medicine and Leipzig University. SMAΔ7 (JAX#00,525) mice were bred to hemizygous Thy1.2-HA-NRG1-III “HANI” mice originated from Max Planck Institute of Experimental Medicine in Göttingen, Germany. SMAΔ7 mice were genotyped as previously described [41] and the *NRG1 Type III* transgene was genotyped using the following primers: forward primer 5'-GGCTTTCTCTGAGTGGCAAAGGACC -3', reverse primer 5'-GTCCACAAATACCCACTTTAGGCCAGC -3'. Investigators were blinded to mouse genotypes during phenotypic assessments, which included daily body weights, righting time and survival [22]. Postnatal day 1 (P1) was defined as the day of birth.

### RNA isolation and RT-qPCR analysis

RNA was isolated using TRIzol reagent (Thermo Fisher Scientific) and RT- qPCR was performed as previously described [22]. Custom Primers used to amplify human *NRG1 Type I* were: forward 5ʹ-GCCAATATCACCATCGTGGAA-3ʹ, reverse 5ʹ-CCTTCAGTTGAGGCTGGCATA-3ʹ, and probe 5ʹ-FAM-CAAACGAGATCATCACTG-MGB-3ʹ. Primers used to amplify human *NRG1 Type III* were: forward 5ʹ-CAGCCACAAACAACAGAAACTAATC-3ʹ, reverse 5ʹ-CCCAGTGGTGGATGTAGATGTAGA-3ʹ, and probe 5ʹ-FAMCCAAACTGCTCCTAAAC-MGB-3ʹ [16]. Primers used to amplify mouse *NRG1 Type I* were Mm00626552_m1 (ThermoFisher) and *NRG1 Type III* Mm01212129_m1 (ThermoFisher).

### Western blot analysis

Western blots were completed as previously described [29]. Proteins were separated by size and transferred onto a 0.2 μm polyvinyl difluoride (PVDF) membrane (ThermoFisher). Antibodies used were rat anti-HA antibody (Millipore Sigma), mouse anti-SMN antibody (BD Transduction Laboratories) and mouse anti-GAPDH (ThermoFisher) antibody.

### Immunohistochemistry

Mice were transcardially perfused with 4% paraformaldehyde (PFA) and post-fixed in PFA. Spinal cords were embedded in warm 5% Agar and serial transverse Sects. (75 μm) were cut on a vibratome. Primary antibodies included anti-ChAT (goat, AB144P, Merck) 1:250 and NRG1 C-terminal antibody, anti-NRG1 (rabbit, SC-348, Santa Cruz) 1:300. Secondary antibodies included the appropriate species-specific antiserum coupled to Alexa488, Cy3 or Cy5 (Jackson labs). Sections were imaged using a SP8 Leica confocal microscope [5] and NRG1 spots in ChAT + MNs from at least 3 animals per genotype were counted using LASX software from z-stack images (complete MN somata at 4 μm intervals in the z axis). Only whole ChAT + MNs that contained the nucleus were included for quantification.

VRs and DRs were sectioned (20 µm thick) on a cryostat (Leica) and stained with primary antibodies against Tuj1 (Chicken IgY, 1:1000), NFH (MsIgG1, 1:1000, Biolegend) and anti-NRG1 (rabbit, 1:400, Santa Cruz). Secondary antibodies included Alexa Fluor (AF) 633 Goat-anti-rabbit (1:500, Invitrogen), AF546 Goat-anti-chicken, (1:1000, Invitrogen), and AF488 Goat-anti-Ms IgG1, 1:200 (Jackson Immunology). Sections were imaged using a Zeiss Confocal Laser Microscope. Neuromuscular junctions (NMJs) were examined in teased myofibers of quadratus lumborus (QL) muscles [22] and labeled using monoclonal mouse anti-SMI 312 (1:1000; Biolegend) and polyclonal rabbit anti-synaptophysin (1:500; Invitrogen) antibodies, followed by AF488 goat anti-mouse IgG1 (1:200; Jackson Immunoresearch Laboratories), AF 633-conjugated goat anti-rabbit secondary antibody (1:1000; Invitrogen), and α-Bungarotoxin AF-555 (1:500; Invitrogen).

### Light and electron microscopy

VRs and DRs were post-fixed using 2% osmium tetroxide and embedded in propylene oxide and EMbed 812 plastic (Electron Microscopy Sciences). Thick Sects. (1 μm) were cut, stained with toluidine blue, and imaged using a Zeiss Axiovision microscope. Area and number of myelinated axons were counted using ZenLite software [22]. For electron microscopy (EM), thin sections were cut at 60–90 nm, placed on bar-less formvar grids, and viewed using a Libra 120 (Zeiss) transmission EM. Consecutive overlapping images were taken at 8000 × or 16,000 × and reconstructed using Adobe Photoshop. The number of axons, axon diameters, and G-ratios were quantified in reconstructed VR images using Zen lite software.

### Serum NF quantification

Cardiac puncture was performed using a 31-gauge syringe for blood collection into a Protein LoBind microcentrifuge tube (500 μl). Serum was isolated and NF-L concentrations were determined using the single molecule array (Simoa) Quanterix platform with NF-Light® (SR-X) kit (Quanterix 103,400) and SR-X instrument [22].

### Electrophysiology

The function of motor axons and NMJs innervating the QL muscle were assessed as previously described [6, 22]. Animals were decapitated, the torso transferred into a dissecting chamber with cold (~ 12 °C) oxygenated (95%O_2_/5%CO_2_) artificial cerebrospinal fluid (aCSF) containing 128.35 mM NaCl, 4 mM KCl, 0.58 mM NaH_2_PO_4_.H20, 21 mM NaHCO_3_, 30 mM D-Glucose, 1.5 mM CaCl_2_.H_2_0, and 1 mM MgSO_4_.7H_2_0. After a laminectomy, the spinal cord was removed, leaving the lumbar level 1 (L1) VR intact in continuity with the QL muscle. MN axons in the L1 VR supplying the QL muscle were stimulated, and the compound muscle action potential (CMAP) was recorded from the muscle using a concentric bipolar electrode. L1 MN axons were stimulated with five stimuli at 1 Hz for peak-to-peak measurements of the maximum CMAP amplitude from five averages.

The extracellular recorded potentials were recorded (DC – 3 kHz, Cyberamp, Molecular Devices) in response to a brief (0.2 ms) stimulation (A365, current stimulus isolator, WPI, Sarasota, FL) of the L1 VR. Recordings were fed to an A/D interface HEKA EPC10/2 amplifier (HEKA Elektronik, Lambrecht/Pfalz, Germany) and acquired with HEKA Patchmaster (HEKA Electronics) amplifier at a sampling rate of 20 kHz. Data were analyzed offline using HEKA Patchmaster (HEKA Electronics). The latency was determined by measuring the time between the beginning of the stimulus artifact and the onset of the CMAP response. The conduction velocity was calculated by the time of the latency minus the time of synaptic transmission, divided by the distance of the stimulating and recording electrode.

### Statistical analysis

Data were expressed as means ± SEM with at least three independent samples per group. Differences between two groups were analyzed by a two-tailed unpaired Welch’s t test or Mann–Whitney for nonparametric analysis, where appropriate, using GraphPad Prism 9. Differences among three of more groups were analyzed with one-way analysis of variance (ANOVA) using Tukey’s post hoc correction. Survival curves were compared using log-rank test.

## Results

### NRG1 expression is reduced in SMA

**Fig. 1:**
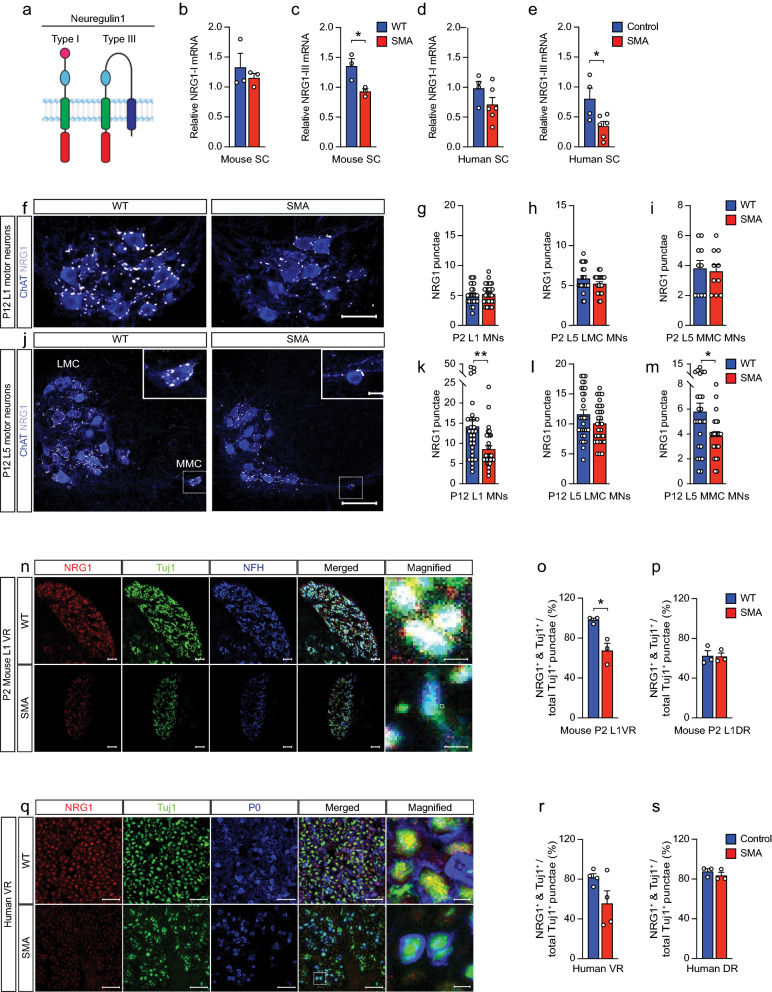
NRG1 expression is reduced in severe SMA mice and patients **A** Schematic of NRG1 protein isoforms, Type I and Type III. **B** Relative *NRG1-I* and **C** *NRG1-III* mRNA expression levels in spinal cords of WT and SMA mice at P1 (n = 3 each). **D** Relative *NRG1-I* and **E** *NRG1-III* mRNA expression levels in spinal cords of controls and SMA Type 1 cases. **F** Representative L1 MNs stained with ChAT (blue) and NRG1 (white) in WT and SMA mice at P12 (scale bar: 50 μm). Quantification of NRG1 punctae on **G** L1 MNs, **H** L5 lateral motor column (LMC) MNs, and **I** L5 medial motor column (MMC) MNs from P2 WT and SMA mice (n = 3 each). **J** Representative LMC and MMC L5 MNs stained with ChAT (blue) and NRG1 (white) in WT and SMA mice at P12 (bottom scale bar: 100 μm and top scale bar: 20 μm.) Quantification of NRG1 punctae on **K** L1 MNs, **L** L5 LMC MNs, and **M** L5 MMC MNs in P12 WT and SMA mice (n = 3 each). **N** Representative confocal images of L1 VRs stained with NRG1 (red), Tuj1 (green), NFH (blue) in WT and SMA mice at P2 (scale bars: 10 μm and magnified scale bar: 1 μm). **O** Percentage of Tuj1 + overlap with NRG1 + over total Tuj1 + punctae of WT and SMA mice at P2 (n = 3 each) in L1 VRs and **P** DRs. **Q** Representative confocal images of human VRs stained with NRG1 (red), Tuj1 (green), P0 (blue) from CNTL 12-02 and SMA 11-01 (scale bar: 20 μm and magnified scale bar: 2.5 μm). **R** Percentage of Tuj1 + overlap with NRG1+ over total Tuj1 + punctae in VRs of controls and SMA Type 1 cases and **S** in DRs of controls and SMA Type 1 cases. Data represents means and SEM. Statistical analysis was performed using unpaired t test. Significance: *p $\le$ .05, **p $<$ 0.01

We examined mRNA expression levels of two isoforms of NRG1: NRG1-III, which is abundant in developing MNs and axons [32], and NRG1-I, which is expressed in Schwann cells during remyelination of peripheral nerves [45] (Fig. 1a). *NRG1-III*, but not *NRG1-1*, was reduced in postnatal day 1 (P1) SMAΔ7 compared to wild type (WT) mouse spinal cords (Fig. 1b,c) as well as in human type I SMA compared to age-matched control spinal cords (Table 1, Fig. 1d,e). As NRG1 is enriched at the post-synaptic terminals of C-bouton synapses [15], we examined NRG1 expression on MNs in P2 and P12 lumbar level 1 (L1) and L5 mouse spinal cords using an antibody that recognizes all NRG1 isoforms. Although the number of NRG1 + punctae on L1 and L5 MNs was unchanged at P2 (Fig. 1g–i), they were reduced on P12 SMA L1 MNs and L5 medial motor column (MMC) MNs (Fig. 1f,j–m). We next examined NRG1 expression in L1 ventral roots (VRs) (containing motor axons) and dorsal roots (DRs) (containing sensory axons) [22]. In contrast to the late loss of NRG1 on MN somata, we observed a decrease in the percentage of Tuj1 positive punctae expressing NRG1 in SMA mouse VRs, but not DRs as early as P2 (Fig. 1n–p). The percentage of axons expressing Tuj1, NF-H, and NRG1 were also decreased in VRs of SMA compared to WT mice at P2 (WT = 64.3 ± 6.7% vs. SMA = 30.2 ± 7.6%, *p* < 0.05). The percentage of Tuj1^+^ punctae expressing NRG1 was variable in human SMA VRs and when taken together did not show a significant decrease relative to controls (*p* = 0.097) (Fig. 1q,r). However, the case with the highest value (SMA14-04) was substantially older at 72 months compared to the other 3 SMA cases, which were all ≤ 7 months old (Table 1). With this older case removed, there was a significant decrease (*p* = 0.007) in the percentage in NRG1 + Tuj1 punctae in SMA VRs compared to controls. No decrease was observed in DRs (Fig. 1p,s). Taken together, these data indicated that NRG1 levels are reduced in SMA MN somata and axons of SMA mice and patients.

### Boosting NRG1-III expression hastens SMA motor axon development

**Fig. 2:**
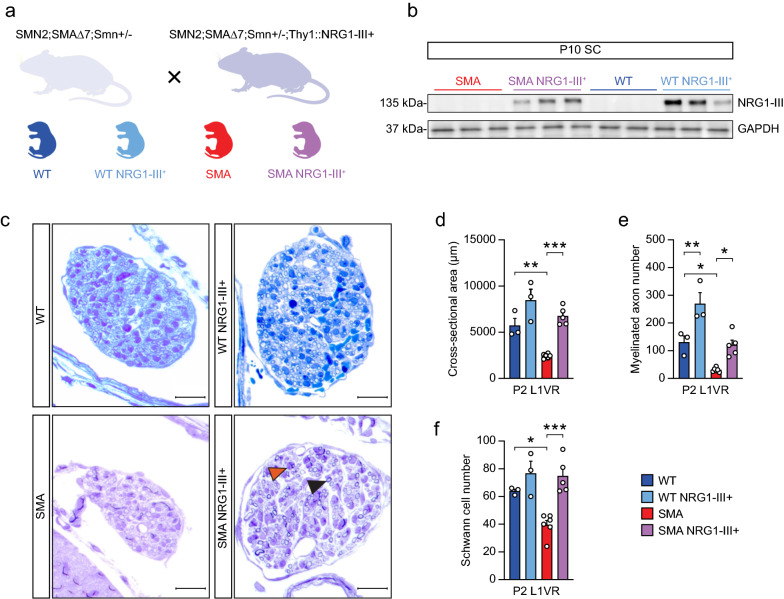
Boosting NRG1-III expression in SMA mice increases VR myelinated axon number **A** Schematic of mouse breeding to generate genotypes: WT, WT NRG1-III + , SMA, and SMA NRG1-III + . **B** Western blot of NRG1-III protein expression in spinal cord protein lysates collected from WT, WT NRG1-III + , SMA, and SMA NRG1-III + mice at P10 (n = 3 each). **C** Representative toluidine blue–stained cross sections of L1 VRs in WT, WT NRG1-III + , SMA, and SMA NRG1-III + of mice at P2 (scale bar: 20 μm). The black arrow indicates a myelinated axon and the orange arrow indicates a Schwann cell nucleus. **D** Cross-sectional area, **E** myelinated axon number and **F** Schwann cell number in L1 VRs from WT (n = 3), WT NRG1-III + (n = 3), SMA (n = 6), and SMA NRG1-III + (n = 5) mice at P2. Data represent means and SEM. Statistical analysis was performed **D**–**F**, using one-way ANOVA. Significance of WT compared to SMA NRG1-III + and WT NRG1-III + compared to SMA not included. Significance: *p $\le$ .05, **p $<$ 0.01, ***p $<$ 0.001

To test whether increased NRG1-III expression would mitigate deficits of axon maturation in SMA mice, we bred SMAΔ7 mice to mice overexpressing N-terminally HA-tagged full-length *NRG1-III* cDNA under control of the neuronal Thy1.2 promoter (Thy1.2-HA-NRG1-III mice) [48] generating mice of the following genotypes: WT (*hSMN2*^+*/*+^*/SMAΔ7*^+*/*+^*/mSmn*^+*/*+^), WT NRG1-III + (*hSMN2*^+*/*+^*/SMAΔ7*^+*/*+^*/mSmn*^+*/*+^*/HA-NRG1-III*), SMA (*hSMN2*^+*/*+^*/SMAΔ7*^+*/*+^*/mSmn*^*−/−*^), and SMA-NRG1-III + (*hSMN2*^+*/*+^*/SMAΔ7*^+*/*+^*/mSmn*^*−/−*^*/HA-NRG1-III*) (Fig. 2a). Western blots from spinal cord lysates isolated from P10 mice demonstrated expression of the HA-NRG1-III protein in both WT NRG1-III + and SMA NRG1-III + mice, but not WT or SMA mice not harboring the *NRG1-III* transgene (Fig. 2b). The expression of NRG1-III did not alter the expression of SMN protein in WT or SMA mouse spinal cords (Additional file 1: Fig. S1).

We examined VRs at the L1 level, where we have previously shown that motor axons are particularly vulnerable to developmental defects [22] (Fig. 2c). SMA VRs were reduced in their cross sectional area and had fewer myelinated axons and Schwann cell nuclei compared to WT VRs (Fig. 2c–f). In WT mice, NRG1-III overexpression increased myelinated axon number by 106%, but only showed a trend (*p* = 0.05) to increased VR area and did not show a change in Schwann cell number (Fig. 2d–f). In contrast, boosting NRG1-III expression in SMA mice increased VR cross sectional area by 177%, the number of myelinated axons by 272%, and the number of Schwann cells by 89%, restoring each of these parameters to levels observed in WT mice not overexpressing NRG1-III (Fig. 2d–f).

**Fig. 3:**
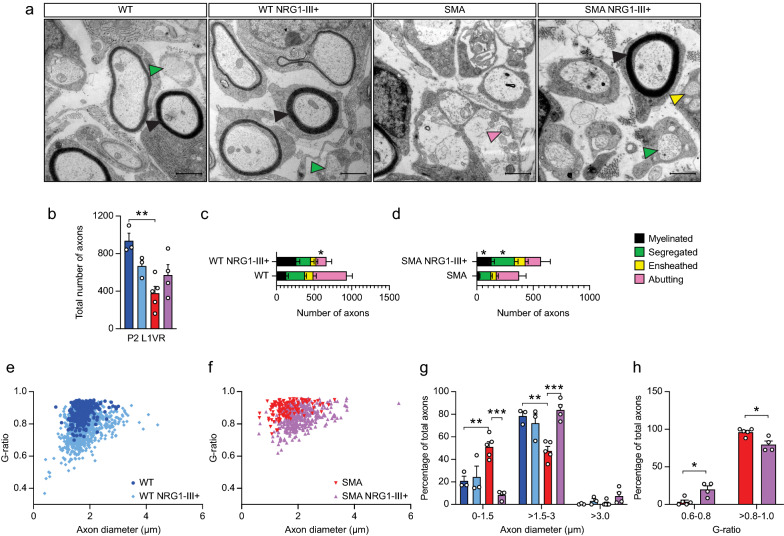
NRG1-III overexpression accelerates several aspects of motor axon development in SMA mice **A** Representative EM images of L1 VRs in WT, WT NRG1-III + , SMA, and SMA NRG1-III + mice at P2 (scale bar: 1 μm). The black, green, yellow, and pink arrows indicate myelinated, segregated, ensheathed and abutting axons, respectively. **B** Quantification of total number of axons in L1 VRs of WT (n = 3), WT NRG1-III + (n = 3), SMA (n = 5), and SMA NRG1-III + (n = 4) mice at P2; separated by axon category for WT and WT NRG1-III + **C** and SMA and SMA NRG1-III + **D** mice. (**E** and** F**) Axon diameters and G-ratios of L1 VRs in WT and WT NRG1-III + **E** and SMA and SMA NRG1-III + **F** mice. **G** Percentage of axons by axon diameter for each genotype. **H** Percentage of axons by different G-ratio categories for SMA and SMA NRG1-III + mice. Data represents means and SEM. Statistical analysis was performed (**B**) and (**H**), using one-way ANOVA; in (**C**), (**D**) and (**G**) using unpaired t test. Significance of WT compared to SMA NRG1-III + and WT NRG1-III + compared to SMA not included. Significance: *p $\le$ .05, **p $<$ 0.01, ***p $<$ 0.001

At the electron microscopy (EM) level, boosting NRG1-III expression did not change the total number of axons in either WT or SMA mice (Fig. 3a,b). Motor axons were further classified based on developmental stage into abutting, ensheathed, segregated and myelinated types as defined previously [22]. In WT mice overexpressing NRG1-III, a decrease in the number of abutting axons was not accompanied by significant changes in other axon types (Fig. 3c). In contrast in SMA mice, a 437% increase in myelinated axon number and a 98% increase in segregated axon number indicated that NRG1-III not only reinstated myelination of axons, but also partially restored the sorting/segregation of axons by Schwann cells into the one-to-one relationships required for myelination (Fig. 3d).

To further characterize the myelinated motor axons, we determined their diameter and myelin thickness. In WT mice, NRG1-III overexpression did not increase the diameter of myelinated motor axons (mean WT = 1.7 µm vs. WT NRG1-III +  = 1.8 µm, *p* = 0.89) (Fig. 3e,g), but did reduce the median G-ratio from 0.89 to 0.78 (*p* = 0.002) indicating thicker myelin sheaths. In SMA mice, NRG1-III overexpression restored median axon diameter (SMA = 1.5 µm vs. SMA NRG1-III +  = 2.0 µm, *p* = 0.002) as well as the percentage of myelinated axons with a diameter between 1.5 and 3 µm to WT levels (Fig. 3f,g). Additionally, while median G-ratio was unchanged, boosting NRG1-III expression increased the percentage of SMA myelinated axons with lower G-ratio = 0.6–0.8 and decreased those with a higher G-ratio = 0.8–1.0 (Fig. 3f,h). These data indicate that in SMA but not WT mice, boosting NRG1-III expression accelerated multiple motor axon developmental steps including radial growth, Schwann cell segregation and myelination.

### NRG1-III overexpression improves neonatal motor axon conduction velocity, but does not prevent axon degeneration

**Fig. 4:**
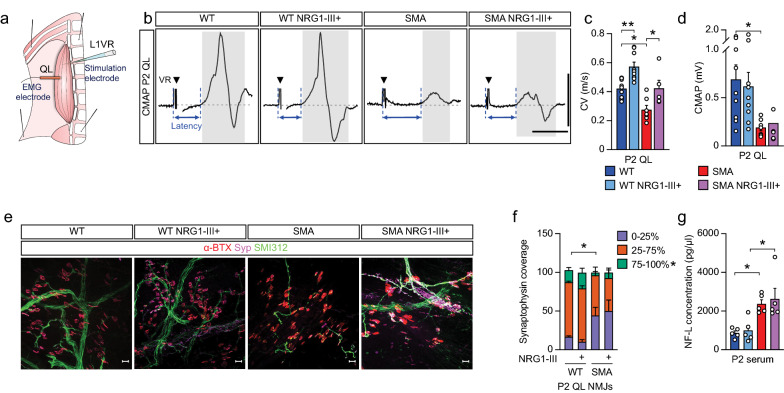
NRG1-III improves neonatal SMA motor axon conduction speed but does not prevent degeneration **A** A schematic of the ex vivo nerve muscle preparations for stimulating L1 VR and recording from the quadratus lumborum (QL) muscle. **B** Representative traces from the QL muscle in WT, WT NRG1-III + , SMA, and SMA NRG1-III + mice at P2. **C** Conduction velocity and **D** CMAP amplitude from the QL muscle of WT (n = 9), WT NRG1-III + (n = 9), SMA (n = 7), and SMA NRG1-III + (n = 5) mice at P2. **E** Representative confocal images of P2 QL muscle NMJs stained with α-bungarotoxin (α-BTX, red), synaptophysin (Syp, purple), and SMI312 (green) (scale bar: 20 μm). **F** NMJ innervation in the L1 QL muscle of WT, WT NRG1-III + , SMA, and SMA NRG1-III + (n = 3 each) mice at P2. **G** Serum NF-L concentration of WT, WT NRG1-III + , SMA, and SMA NRG1-III + (n = 5 each) mice at P2. Data represents means and SEM. Statistical analysis was performed in (**C**, **D**) and (**G**) using one-way ANOVA; and in (**F**) using unpaired t test. Significance of WT compared to SMA NRG1-III + and WT NRG1-III + compared to SMA not included. Significance: *p $\le$ 0.05, **p $<$ 0.01

To assess whether the improvements in early SMA motor axon development were associated with alterations in function, we performed electrophysiology using ex vivo nerve muscle preparations (Fig. 4a,b). Consistent with prior observations, the conduction velocity of SMA motor axons was reduced compared to WT mice (Fig. 4c). In both WT and SMA mice, NRG1-III overexpression increased conduction velocity with SMA mice reaching similar levels to WT mice (Fig. 4c). Despite this improvement, which likely relates to the increased number, diameter and myelin thickness of myelinated SMA motor axons, the compound muscle action potential (CMAP) amplitudes, which represent the summated voltage responses from the individual muscle fiber action potentials generated by the most rapidly conducting axons, was not increased by NRG1-III overexpression in SMA mice (Fig. 4d).

Unchanged CMAP amplitudes in SMA NRG1-III overexpressing mice suggest an ongoing inability of motor axons to stimulate a muscle action potential. We thus examined the innervation status of neuromuscular junctions (NMJs) in the quadratus lumborus (QL) muscle using immunohistochemistry. As expected, there was an increase in the number of denervated NMJs in the QL of SMA compared to WT mice, but NRG1-III overexpression did not alter these denervation percentages (Fig. 4e,f) nor did it suppress levels of serum NF-L in SMA mice (Fig. 4g). Together these data indicate that NRG1-III overexpression does not suppress degeneration of the distal motor axon in SMA mice.

### NRG1-III does not provide sustained benefit to SMA motor axons

**Fig. 5:**
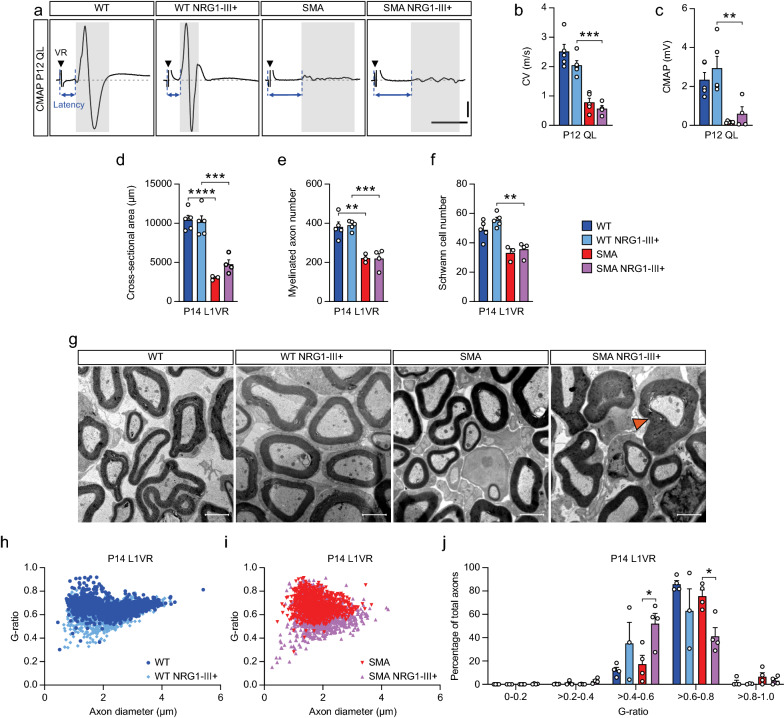
NRG1-III overexpression does not provide sustained improvement to SMA motor axon morphology or function **A** Representative EMG traces from the QL muscle in P12 mice. **B** Conduction velocity from the QL muscle of WT (n = 5), WT NRG1-III + (n = 5), SMA (n = 5), and SMA NRG1-III + (n = 4) mice at P12. **C** CMAP amplitude from the QL muscle of WT (n = 5), WT NRG1-III + (n = 5), SMA (n = 6), and SMA NRG1-III + (n = 4) mice at P12. **D** Cross-sectional area, **E** myelinated axon number and **F** Schwann cell number of L1 VRs in WT (n = 5), WT NRG1-III + (n = 5), SMA (n = 3), and SMA NRG1-III + (n = 4) mice at P14. **G** Representative EM images of L1 VRs in WT, WT NRG1-III + , SMA, and SMA NRG1-III + mice at P14 (scale bar: 2 μm). The orange arrow indicates a large myelin sheath. (**H** and** I**) Axon diameters and G-ratios of L1 VRs in WT and WT NRG1-III + **H** and SMA and SMA NRG1-III + **I** P14 mice. **J** Percentage of axons by axon diameter in L1 VR of WT (n = 4), WT NRG1-III + (n = 3), SMA (n = 4), and SMA NRG1-III + (n = 4) mice at P14. Data represents means and SEM. Statistical analysis was performed in (**B**)–(**F**) using one-way ANOVA; and in (**J**) using unpaired t test. Significance of WT compared to SMA NRG1-III + and WT NRG1-III + compared to SMA not included. Significance: *p $\le$ .05, **p $<$ 0.01, ***p $<$ 0.001, ****p $<$ 0.0001

In order to assess the longer-term consequences of NRG1-III overexpression on WT and SMA mice, we characterized mouse survival, weight, and time to right (Additional file 2: Fig. S2). Boosting NRG1-III expression did not ameliorate deficits in SMA mice; rather survival was reduced by NRG1-III overexpression (*p* = 0.006). We also examined the electrophysiology and structure of motor axons at P12 compared to P2 (Figs. 4b–d and 5a–c). CVs increased by 497% (*p* = 0.001) and 257% (*p* < 0.001) between P2 and P12 in WT and WT NRG1-III + mice, respectively (Figs. 4c and 5b). This increase of CV was associated with decreases in G-ratios [25% in WT (p < 0.001) and 20% in WT NRG1-III + (*p* = 0.029)] and a 32% increase in myelinated axon diameter in WT mice (*p* < 0.001) (Figs. 3e and 5h). Between P2 and P12, CMAP amplitudes increased by 239% (*p* = 0.009) in WT mice and 376% (*p* = 0.017) in WT NRG1-III + mice (Figs. 4d and 5c) corresponding with a 193% increase in myelinated axon number in WT mice (*p* < 0.001) (Figs. 2e and 5e). In contrast, SMA and SMA NRG1-III + mice showed no increases in CMAP amplitudes between P2 and P12 likely due to ongoing axon degeneration mitigating any developmental gains. While at P2, NRG1-III was able to restore myelinated axon number, myelinated axon diameter, and conduction velocity in SMA mice to WT levels, further developmental gains in axonal diameter and conduction velocity did not occur in SMA NRG1-III + mice between P2 and P14 (Fig. 5d–f). NRG1-III overexpression did increase the percentage of SMA axons with thick myelin sheaths (G-ratio > 0.4–0.6), but in the absence of an increase in axon diameter, this was not sufficient to increase CV (Fig. 5g,i,j).

## Discussion

In the most common severe SMA patient group, degeneration of the most immature motor axons is most evident neonatally and may even begin in utero [22]. The molecular mechanisms downstream of SMN deficiency that underlie slowed axon development and degeneration are unknown. Here, we explored the role of NRG1-III, levels of which are a principal determinant of peripheral axon Schwann cell ensheathment and myelination. We demonstrated that NRG1 expression is reduced in both severe human and mouse SMA spinal cords and VRs. Supporting the conclusion that NRG1-III directly contributes to retarded axon development in SMA, boosting NRG1-III expression accelerated several aspects of SMA motor axon development including Schwann cell number, segregation, myelination, and radial growth. These morphological changes have functional consequences accelerating motor axon conduction velocity neonatally.

In the neuromuscular system, NRG1-III is expressed at high levels by MNs during development [17, 32] and is localized to the surface of their axons regulating the differentiation and survival of Schwann cells. A minimal threshold of NRG1-III expression by individual axons signals the Schwann cell cytoplasm to ensheath it [47]. Subsequent high expression levels by larger axons stimulates segregation into a 1:1 relationship with Schwann cells as well as formation of myelin sheaths [33, 47] that enable saltatory conduction. In contrast, lower levels of NRG1-III expression by autonomic or small sensory axons leads to ensheathment of multiple unmyelinated axons by a single Schwann cell together in a Remak bundle [47]. We have previously shown in severe human SMA mouse VRs at the time of autopsy and in L1 VRs at embryonic and early postnatal time points in SMAΔ7 mice that there is an excess of axons with immature morphologies including the most immature abutting axons within single Schwann cell pockets, and reduced acquisition of myelinated axons [22]. Given this phenotype, it is not surprising to observe reduced NRG1 expression in a proportion of SMA axons. The reduction of *NRG1-III* mRNA levels in the spinal cord is consistent with this arising at least in part from reduced transcription and/or impaired mRNA processing. This could be directly caused by SMN protein deficiency as SMA is required for maintaining the fidelity of spliceosome small nuclear ribonucleoproteins (snRNPs) and NRG1 transcripts undergo extensive alternative splicing [7]. Alternatively, reduced NRG1-III expression may be triggered as a downstream cellular consequence of SMN deficiency such as impaired SMA MN firing [14, 30]. NRG1 type I and IV expression have been shown to be activated by neuronal activity in primary cortical neurons [27]. Consistent with the conclusion that NRG1-III deficiency directly contributes to poor neonatal axon development in SMA, boosting NRG1-III expression accelerated L1 VR axon-Schwann cell developmental interactions including axon segregation and myelination with associated increased VR size, Schwann cell and myelinated axon number. Interestingly, these improvements in myelination were also associated with an increase in axon diameter in SMA NRG1-III mice, which might be secondary to the influence of increased myelination on the trafficking and post-translational modifications of NFs, whose assembly determines axonal radial diameter [10, 18, 37]. Together these pathological changes increased axon conduction velocity at P2. Unfortunately, despite the critical role for Schwann cells in providing neurotrophic and metabolic support to axons [4, 38], the developmental improvements in the relationships between SMA Schwann cells and motor axons were not sufficient to prevent distal axon degeneration at SMA NMJs and consequently, there was no improvement in the CMAP amplitudes.

In addition to its localization to axons, NRG1 is also present at cholinergic synapses including the postsynaptic terminal of C-terminal boutons on MN somata and at NMJs. A previously published study reported a transient increase in NRG1 positive C terminal boutons at P1 in SMAΔ7 compared to WT mice and equivalent numbers at P7 and P15 [15], however the specific lumbar spinal level examined was not defined in this study. Here, we observed a late loss of NRG1 + MN punctae on L1 and L5 medial column MNs, but not L5 lateral column MNs corresponding to those distributions known to be susceptible to axonal degeneration in this model [22]. Loss of NRG1 + punctae intensity on MNs has also been previously observed in the setting of axotomy and in ALS mice [15, 43]. C-terminal boutons are postulated to play an important role in driving MN activity [34, 50]. Whether this is an important contributor to the altered MN firing that has been described in SMA mice [6, 14] will require further study. At the NMJ, NRG1-III signaling is not required for NMJ formation [11, 19], but it has been reported to regulate the behavior of terminal Schwann cells. Neonatal transgenic mice overexpressing NRG1-III have been shown to have an increased number of terminal Schwann cells and accelerated removal of polyneuronal innervation at the NMJ perhaps via enhanced phagocytic activity of these cells [23]. Of note, SMA mice have been reported to have a reduced number of terminal Schwann cells together with a reduced rate of polyneuronal innervation removal [24]. NRG1-III overexpression in the SMA background may have accelerated NMJ pruning events mitigating the positive developmental effects seen at more proximal motor axons.

This work provides a proof-of-principle that SMA motor axon developmental pathologies can be mitigated by molecular strategies independent of increasing SMN expression. NRG1-III has been explored as a therapeutic strategy in preclinical models of genetic neuropathy [2] and in ALS, [35, 36] the later using adeno-associated virus vectors expressing NRG1-III. Although a potential therapeutic strategy in several diseases, successful translation of NRG1-III to humans will require advances in targeted delivery strategies to prevent untoward effects as NRG1 is also widely expressed in the brain. Older Thy1.2-HA-NRG1-III mice develop cortical synaptic dysfunction with associated anxiety-like behaviors [1]. Such untoward effects may have contributed to the poor behavioral and survival outcomes of older SMA mice transgenically overexpressing NRG1-III seen here. Further work is needed to identify methods to deliver NRG1-III specifically to MNs and to assess outcomes in SMA mouse models when NRG1-III is delivered together with current SMN induction therapies. While our study indicates that NRG1-III is one molecular determinant of axon maldevelopment in SMA, further characterization of the molecular mechanisms underlying impaired development and degeneration of SMA motor axons is urgently needed. Our previous work indicates that these events are largely MN cell autonomous as accelerated motor axon development was seen in mice with increased SMN expression in MNs, but not in mice with increased SMN expression in Schwann cells or muscle [22]. Peripheral axon development depends on multiple molecular mediators expressed specifically by Schwann cells or by axons such as adhesion molecules, components of the extracellular matrix, Notch signaling components and Nectin proteins [12, 46]. Most of the mediators identified to date are expressed by Schwann cells with those expressed by axons remaining largely unknown. Continued unbiased molecular phenotyping of developing WT and SMA motor axons is required to identify such neuronally expressed factors.

Strategies to accelerate SMA motor axon development and prevent degeneration promise to be complementary to existing disease modifying therapeutics, which increase SMN expression, but vary in their therapeutic efficacy. Here we identify NRG1-III as a key molecular determinant of impaired motor axon segregation and myelination in SMA. Furthermore, we demonstrate that boosting NRG1-III expression accelerates several steps of SMA neonatal motor axon maturation. This work illustrates the potential of targeting developmental pathways downstream of SMN protein deficiency as a means to ameliorate aspects of disease pathology that cannot be reversed by postnatal SMN protein induction alone.

## Supplementary Information

**Additional file 1**: **Fig. S1.** SMN protein expression in WT and SMA mice overexpressing NRG1-III A SMN protein expression in representative western blots of spinal cord tissues collected from WT (n = 3), WT NRG1-III+ (n = 3), SMA (n = 3), and SMA NRG1-III+ (n = 2) mice at P10. B Quantification of relative SMN expression in western blot in (A).

**Additional file 2**: **Fig. S2.** NRG1-III overexpression fails to improve motor behavior and survival of SMA mice (A–C) Survival, A weight, B and righting time C of WT (n = 9), WT NRG1-III+ (n = 15), SMA (n = 18), and SMA NRG1-III+ (n = 12) mice. Data represents means and SEM. Statistical analysis was performed using log-rank test in (A). Significance: ***p < 0.001.

## Data Availability

All data generated and/or analyzed during this study are included in this published article.
